# Structure and Activity of a Novel Archaeal β-CASP Protein with N-Terminal KH Domains

**DOI:** 10.1016/j.str.2011.03.002

**Published:** 2011-05-11

**Authors:** Ana P.G. Silva, Maria Chechik, Robert T. Byrne, David G. Waterman, C. Leong Ng, Eleanor J. Dodson, Eugene V. Koonin, Alfred A. Antson, Callum Smits

**Affiliations:** 1 York Structural Biology Laboratory, Department of Chemistry, University of York, York YO10 5YW, United Kingdom; 2 Structural Studies Division, MRC Laboratory of Molecular Biology, Hills Road, Cambridge CB2 0QH, England; 3 National Center for Biotechnology Information, National Institutes of Health, Bethesda, MD 20894, USA

## Abstract

MTH1203, a β-CASP metallo-β-lactamase family nuclease from the archaeon *Methanothermobacter thermautotrophicus*, was identified as a putative nuclease that might contribute to RNA processing. The crystal structure of MTH1203 reveals that, in addition to the metallo-β-lactamase nuclease and the β-CASP domains, it contains two contiguous KH domains that are unique to MTH1203 and its orthologs. RNA-binding experiments indicate that MTH1203 preferentially binds U-rich sequences with a dissociation constant in the micromolar range. In vitro nuclease activity assays demonstrated that MTH1203 is a zinc-dependent nuclease. MTH1203 is also shown to be a dimer and, significantly, this dimerization enhances the nuclease activity. Transcription termination in archaea produces mRNA transcripts with U-rich 3′ ends that could be degraded by MTH1203 considering its RNA-binding specificity. We hypothesize that this nuclease degrades mRNAs of proteins targeted for degradation and so regulates archaeal RNA turnover, possibly in concert with the exosome.

## Graphical abstract

**Graphical Abstract:**

**Figure:**
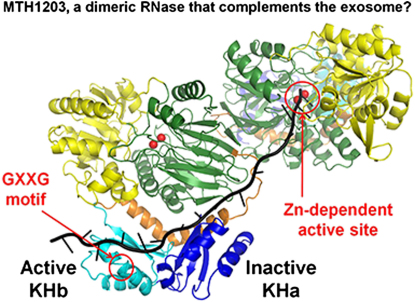

**Highlights:**

► MTH1203 combines RNA-processing and binding domains, unique to archaea ► MTH1203 is a Zn-dependent β-CASP RNase that functions as a dimer ► The MBL domain of MTH1203 binds U-rich sequences with a K_d_ in the micromolar range ► We propose MTH1203 acts with the proteasome degrading mRNAs of aberrant proteins

## Introduction

RNA processing in archaea shares more functional features with the respective processes in bacteria, in contrast to archaeal transcription that, in many respects, resembles transcription in eukaryotes (Andersson et al., 2006; Bini et al., 2002; Olsen and Woese, 1997). The macromolecular complexes that are responsible for degrading cellular RNAs, the degradosome in bacteria and the exosome in archaea and eukaryotes, share similar architectures and are composed of a similar number of RNA-binding and exonuclease modules; however, the archaeal exosome is most closely related to its counterpart in eukaryotes (Houseley et al., 2006). Most archaeal mRNAs appear to have short half-lives, which might reflect the necessity to quickly reprogram gene expression in response to rapid changes in the environment (Andersson et al., 2006). In hyperthermophilic archaea, mRNA decay is stimulated by polyadenylation and mediated by the exosome (Portnoy and Schuster, 2006). In contrast, in halophilic archaea, which lack the exosome and therefore polyadenylation stimulated degradation, an exonuclease homologous to bacterial RNase R has been shown to be responsible for mRNA degradation (Portnoy and Schuster, 2006). Additional nucleases might be involved in the regulation of archaeal RNA degradation and comparative genome analysis can help elucidate differences between the degradation machineries of Archaea that possess the exosome, and those that lack it.

The genes encoding exosome subunits in several archaeal genomes have been predicted in a comparative genomic study using the genomic context and in-depth protein sequence analysis (Koonin et al., 2001). This study also identified additional genes organized in partially conserved predicted operons as possible components of the archaeal exosome. One of these genes encodes a putative ribonuclease (RNase) of the metallo-β-lactamase family that is present in almost all archaeal genomes and is always correlated with a gene encoding a proteasome catalytic subunit. It has been proposed that this putative RNase could be a link between the functions of the exosome and the proteasome (Koonin et al., 2001). The gene encoding this enzyme is present only in archaeal genomes and its function, including its potential association with the exosome, has not been characterized. MTH1203 is the ortholog of this metallo-β-lactamase RNase in the thermophilic archaeon *Methanothermobacter thermautotrophicus*.

MTH1203 belongs to the metallo-β-lactamase (MBL) superfamily (Aravind, 1999; Neuwald et al., 1997). This superfamily includes oxidoreductases, hydrolases that cleave thiol-ester, phosphodiester and sulphuric-ester bonds, and metallo-proteins that cleave β-lactam compounds. Although the 6000 members of this superfamily have low sequence homology, they share a similar overall fold. The MBL domain consists of an αβ/βα sandwich with two β sheets flanked by five solvent exposed α helices. All MBL enzymes contain a metal-dependent active site on one side of the β sheet sandwich that can accommodate one or two zinc ions. Histidine and aspartic acid residues coordinate the zinc ions and are conserved within five distinct motifs (Aravind, 1999; Bebrone, 2007; Garau et al., 2005).

MTH1203 is a member of the β-CASP (metallo-β-lactamase-associated CPSF-73 Artemis SNM1/PSO2) family, a group within the MBL superfamily that comprises enzymes involved in DNA and RNA processing. The signature β-CASP or clamp domain is inserted into the MBL domain and may be involved in substrate recognition (Callebaut et al., 2002; Ishikawa et al., 2006). In addition, sequence analysis predicts that MTH1203 and its archaeal orthologs possess an RNA-binding KH domain at the N terminus, with the conserved glycine-rich GXXG motif that is characteristic of the majority of KH domains (Adinolfi et al., 1999; Grishin, 2001). Nucleic acids are bound in a single stranded conformation within a cleft across one face of the KH domain (Valverde et al., 2008).

Five nonarchaeal β-CASP protein structures have been determined to date: TTHA0252 and RNase J, both functional counterparts of RNase E from *Thermus thermophilus* (de la Sierra-Gallay et al., 2008; Ishikawa et al., 2006); the human protein CPSF-73 and CPSF-100 from yeast, both involved in the maturation of eukaryotic pre-mRNAs (Mandel et al., 2006); and EF2904, a hypothetical protein from *Enterococcus faecalis* (PDB ID 2az4). These structures all contain the MBL and β-CASP domains but no distinct RNA-binding modules.

Here, we report the crystal structure of an archaeal β-CASP protein from *Methanothermobacter themoautotrophicus*. We show that MTH1203 contains not one but two KH domains associated with the MBL and the β-CASP domains, as recently reported for orthologs from *Pyrococcus horikoshii* (Nishida et al., 2010) and *Methanosarcina mazei* (Mir-Montazeri et al., 2011). This novel domain organization was analyzed and compared with the available β-CASP protein structures. We predict that MTH1203 functions as a dimer on the basis of our oligomerization analysis and the conservation of the dimer arrangement in orthologous proteins. The interaction of MTH1203 with the exosome was assessed using SEC-MALLS experiments and a pull-down assay. The predicted RNA-binding ability of MTH1203 and its nuclease activity were confirmed in vitro.

## Results

### Sequence Analysis

**Figure 1:**
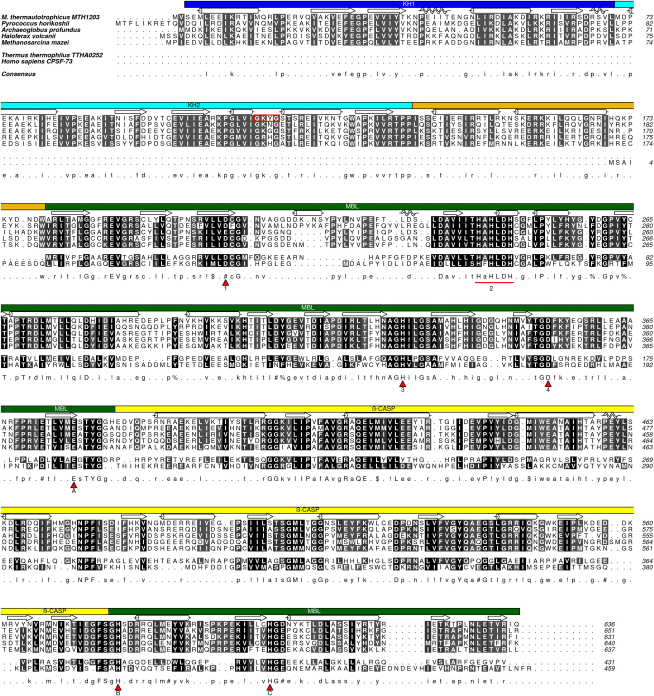
Sequence Alignment of MTH1203 and Its Archaeal Orthologs with Human CPSF-73 and *Thermus thermophilus* TTHA0252 The GXXG motif of the KH fold is highlighted by a red box. The conserved motifs of β-CASP proteins are labeled beneath the consensus. The secondary structure assignment and domain structure is that of the MTH1203. Sequences were aligned using ClustalW (Thompson et al., 1994) and formatted with Aline (Bond and Schuttelkopf, 2009).

Examination of the arCOG database (Makarova et al., 2007) and additional BLAST searches indicate that orthologs of MTH1203 are represented in all sequenced archaeal genomes with the apparent sole exception of the highly reduced genome of *Nanoarchaeum equitans*. Analysis of the MTH1203 amino acid sequence revealed the presence of three domains. At the N terminus, the predicted RNA-binding KH domain is identified by the conserved motif GXXG (Valverde et al., 2008) (residues 114–117, Figure 1). The central region of the protein sequence (residues 188–356) confidently aligns with MBL proteins and shows substantial sequence conservation. Residues Asp208, His242-His247 (HAHLDH), His329 and Asp352 correspond to motifs 1, 2, 3, and 4 of the MBL superfamily, respectively. The C-terminal region (residues 421–545 and 562–605) constitutes the β-CASP or clamp domain (Ishikawa et al., 2006; Mandel et al., 2006), where the characteristic β-CASP motifs A, B and C correspond to residues Glu377, His579, and His603, respectively. MTH1203 is likely to be specific for RNA substrates given that motif C contains a histidine residue whereas DNA-processing enzymes of the β-CASP family contain valine in this position (Callebaut et al., 2002). Thus, sequence analysis suggests that MTH1203 is a β-CASP family member involved in RNA metabolism as this protein is predicted to bind and degrade RNA.

### Overall Structure

The best diffracting crystals were obtained after proteolytic removal of the His-tag and grew in 24 hr in the space group P4_1_2_1_2. The MTH1203 crystal structure was solved by molecular replacement at a resolution of 3.1 Å (Table 1), using X-ray data collected at the zinc absorption peak. A homolog of MTH1203, human CPSF-73 that shares 31% sequence identity over 421 residues of the MBL and β-CASP domains, was used as a search model. Assignment of methionines and model validation were assisted by anomalous data collected from a seleno-methionine protein crystal.

**Figure 2:**
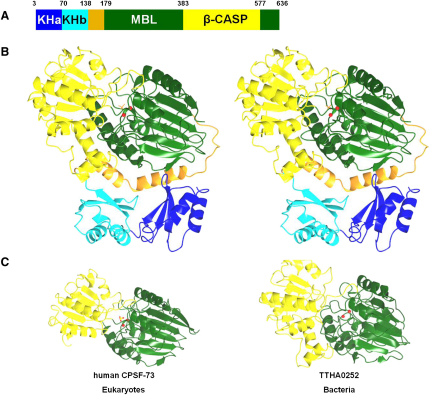
Structure of MTH1203 and Its Homology with β-CASP Proteins from Different Kingdoms (A) Domain organization. Two N-terminal domains with a KH fold (blue and cyan) are connected by a linker segment (orange) with the central metallo-β-lactamase domain (green) followed by the β-CASP clamp domain (yellow). (B) Ribbon diagram of MTH1203 (PDB ID 2ycb) shown in stereo and colored as in (A). (C) Ribbon diagrams of the human CPSF-73 (PDB ID 2i7t) and the bacterial RNase TTHA0252 (PDB ID 2dkf). Red spheres correspond to Zn ions; a phosphate molecule in MTH1203 and a sulfate molecule in CPSF-73 are shown in sticks. The colors of the domains are the same as in (A). See also Figure S1.

The MTH1203 protein has a tripartite architecture consisting of the two N-terminal KH domains (KHa and KHb), the central MBL domain and the C-terminal β-CASP domain (Figure 2A). The two molecules found in the asymmetric unit are essentially identical and superpose with a root mean square deviation of 0.65 Å. Two zinc ions are observed in the active site which is situated at the bottom of a cleft in the MBL domain (Figure 2B). The most significant difference between this structure and the determined β-CASP structures from bacteria and eukaryotes is the presence of an N-terminal RNA-binding module in addition to the catalytic core. Although sequence analysis predicted that MTH1203 contains a single KH domain at the N terminus, the structure revealed two consecutive KH domains. The N-terminal KH domain (KHa, residues 1–70) was not detected by sequence analysis because it lacks the signature GXXG motif. Conserved regions in the KHa domain, in contrast to the poor conservation observed in the interdomain linkers from a variety of archaeal orthologs, suggest that the KHa domain is functionally relevant. The MTH1203 protein contains two additional α helices (α6 and α7) and a flexible loop (residues 139–179, colored in orange in Figure 2) that are absent in β-CASP proteins from bacteria and eukaryotes. These helices connect the KHb domain to the MBL core and are conserved in the archaeal orthologs of MTH1203 (Figure 1).

### The Metallo-β-Lactamase Domain

This α/β fold is highly conserved in MBL proteins including MTH1203. It consists of the central region (residues 180–383) between the N-terminal KH2 domain and the β-CASP domain, and the C-terminal region (residues 578–636), which folds back to contribute three β strands and two α helices to the MBL domain (colored green in Figure 2). The active site is located on one side of this β sandwich.

### The β-CASP/Clamp Domain

The β-CASP domain is formed by residues 384–577 of MTH1203 (colored in yellow in Figure 2). Similar to other β-CASP family members, the characteristic A, B, and C motifs of MTH1203 are part of the MBL domain (not β-CASP domain). The active site pocket is covered by flexible loops from the MBL and β-CASP domains. These loops may rearrange or relative movements between the MBL and β-CASP domains might enable the RNA substrate to access the active site. The width of the active site in MTH1203 is around 10 Å, similar to that of TTHA0252, implying that only single-stranded RNA segments can be accommodated.

### MTH1203 Contains a Conserved Metal-Bound Active Site

**Figure 3:**
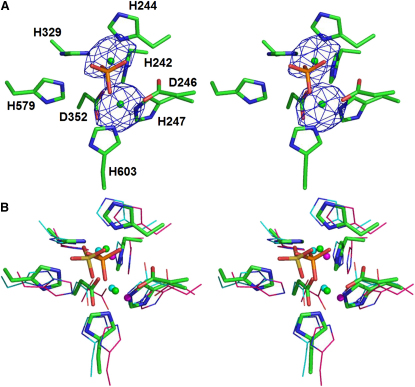
Metal-Containing Active Site of the MBL Domain (A) Anomalous difference maps calculated using the peak data collected at the zinc absorption edge and contoured at 5 σ. (B) Superposition of the MBL active sites of MTH1203 (green), human CPSF-73 (cyan), and bacterial TTHA0252 (magenta). The spheres correspond to the Zn atoms while the MTH1203 phosphate and the CPSF-73 sulfate molecules are shown in orange and yellow sticks, respectively.

The active site of MTH1203 is located at one edge of the β sandwich in the MBL domain and contains two zinc ions (Figure 3A). The positions of zinc atoms at this site were confirmed by anomalous difference maps. The two zinc atoms are coordinated in an octahedral geometry by the bound phosphate and the conserved residues His242, His244, Asp246, and His247 from motif 2, His329 (motif 3), Asp352 (motif 4), His579 (motif B), and His603 (motif C) located in the MBL domain, similar to the active site structures of CPSF-73 and TTHA0252 (Figure 3B). Motifs 1 (Asp208) and A (Glu377) position His247 and His579/His603, respectively, for zinc coordination. No water molecules were observed in the active site due to the resolution of the data. A phosphate group present in the active site is a good mimic for the phosphate group of a nucleotide ready to be hydrolysed. This is similar to a sulfate group and an activated water molecule, suitable for a nucleophilic attack to initiate hydrolysis, observed in the active site of human CPSF-73 (Mandel et al., 2006).

### MTH1203 Contains Two KH Domains at the N Terminus

**Figure 4:**
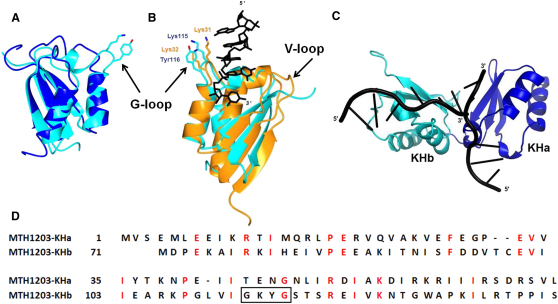
KH Domains: Structures and Functional Implications (A) Superposition of the KHa (blue) and KHb (cyan) domains. (B) The KHb domain, rotated 180° around a vertical axis with respect to (A), is superposed with the Poly(C)-Binding Protein-2 KH1 domain (PCBP2-KH1, orange) complexed with 5′-AACCCU-3′ RNA segment (PDB ID 2py9; black). The G-loop of both KH domains and the variable loop (V-loop) of the PCBP2-KH1 are labeled. The side chains of K31 and K32 of PCBP-2 as well as K115 and Y116 of MTH1203 are highlighted. (C) Models of MTH1203 KHa and KHb domains in complex with RNA segments (black) generated by superposition with the PCBP2-RNA complex. (D) Sequence alignment of the MTH1203 KHa and KHb domains. The black box highlights the conserved GXXG RNA-binding motif found in the KHb of MTH1203. Side chains of the Lys and Tyr residues of this loop are shown in (A).

The N-terminal region of MTH1203 contains the KHa and KHb domains comprised of residues 3–70 and 71–138, respectively. The rmsd between these two domains is only 1.4 Å (overall sequence identity 23%) if the glycine-rich RNA-binding segment of KHb (112–120) is excluded from the comparison. The major structural differences between the two domains are in the glycine-rich segment (Figure 4A). Both KHa and KHb are type II KH domains (Valverde et al., 2008), which are commonly found in prokaryotes and possess an αββααβ fold (Backe et al., 2005; Lewis et al., 1999; Shin et al., 2003).

### Only KHb Is Predicted to Bind Nucleic Acids

To examine the potential interactions of the tandem KH domains of MTH1203 with RNA, models of the KH domains bound to RNA were created by superposition with the structure of the first KH domain from human Poly(C)-Binding Protein-2 (PCBP2) complexed with AACCCU RNA (Du et al., 2007) (PDB ID 2py9) (Figure 4B). Although the KH domain of PCBP2 is a type I KH domain, the rmsd between the Cα atoms of superposed helix-turn-helix segments that contain the conserved GXXG motif (residues 106–128 and 22–44 in MTH1203 and PCBP2, respectively) is only 1.7 Å. The superposition suggests that Lys115 and Tyr116 of MTH1203 could interact with RNA similarly to the key RNA-binding residues in PCBP2 (Lys31 and Lys32). Interestingly, the RNA-binding site of KHb is distant to the active site of the MBL domain, being separated by ∼42 Å with no significant positively charged regions observed on the molecular surface between the two domains (see Figure S1 available online).

The KHa domain was not identified by sequence analysis. A model of the KHa/RNA complex based on homology to KHb shows that the predicted RNA-binding region of KHa is accessible to RNA (Figure 4C). However, this domain lacks the GXXG motif that is essential for RNA binding (Lin et al., 1997; Siomi et al., 1994), and the conformation of its helix-turn-helix structure, crucial for RNA binding, is different suggesting that the KHa domain does not bind RNA.

### MTH1203 Binds U-Rich ssRNA

**Figure 5:**
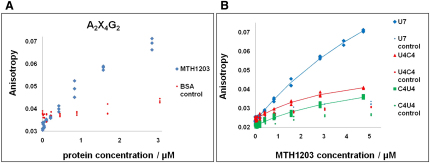
MTH1203 Binds Single Stranded U-Rich Sequences The change in fluorescence polarization anisotropy of fluoresceine-labeled RNAs was measured using increasing MTH1203 concentrations with BSA as a negative control. (A) Titrations performed using the degenerate sequence 5′-AAXXXXGG-3′. (B) Titration of U_7_, U_4_C_4_ and C_4_U_4_ indicated an interaction with a calculated K_d_ of 9.2, 3.6, and 5.2 μM, respectively. Calculation of the dissociation constants using Scientist (Micromath) indicated a good fit of the data, with the associated residual errors of 4% or less (data not shown). Data points correspond to multiple measurements from the same titration. See also Figures S2 and S3.

RNA-binding experiments were performed using polarization anisotropy. The specificity of RNA-binding by MTH1203 is unknown. Therefore, RNA-binding experiments were performed in three stages: initially to verify the ability of MTH1203 to bind ssRNA fragments, subsequently to determine if MTH1203 interacts with selected RNA sequences and finally to assess the RNA binding to the KH domains in MTH1203. An initial experiment using the degenerate sequence A_2_X_4_G_2_, a pool of RNAs containing all 256 possible sequences, indicated that MTH1203 binds ssRNA (Figure 5A). Each binding experiment was compared to the corresponding control with BSA, which does not bind nucleic acids (Curry et al., 1999). An experimental dissociation constant could not be calculated for the A_2_X_4_G_2_ oligonucleotide because the concentration of the subpopulation of sequences that bound MTH1203 is unknown. Further experiments were performed to determine if MTH1203 interacts with selected specific sequences. The assays showed that MTH1203 does not bind A_7_, C_7_ or G_7_ oligonucleotides (Figure S2A) as the anisotropy values throughout the titrations were similar to the negative control experiments with BSA. In contrast, MTH1203 interacted with the U_7_, U_4_C_4_, C_4_U_4_, and A_4_G_4_ sequences (Figure 5B; Figure S2B) with calculated dissociation constants of 9.2, 3.6, 5.2, and 5.3 μM, respectively.

Finally, RNA-binding experiments were performed using only the KH domains of MTH1203, either alone or in tandem (KHa, KHb, and KHa-b). These domains were all shown to be folded in solution by CD spectroscopy (Figure S3A). An initial pull-down assay of these three constructs was performed with the degenerate A_2_X_4_G_2_ oligonucleotide. The strongest RNA-binding activity was detected with the KHb domain. The KHa-b construct showed 44% of the RNA-binding of KHb, whereas KHa bound only 25% (Figure S3B). This result supports the sequence-based prediction that KHb and KHa are the active and inactive RNA-binding domains, respectively. Fluorescence polarization anisotropy experiments were subsequently performed for these constructs using the defined RNA oligonucleotides; however, no change in the polarization anisotropy was observed, meaning that the KH domains do not bind any of the defined RNA sequences tested (data not shown).

These results demonstrate that MTH1203 binds ssRNA with affinities in the micromolar range. The lower dissociation constant for U_4_C_4_ and C_4_U_4_ than for U_7_ suggests that the protein binds U-rich sequences tighter than poly(U) sequences. Additionally, as MTH1203 appears to bind A_4_G_4_ but not A_7_ or G_7_, the protein might recognize the sequence AG. However, it is likely that these interactions involve the MBL domain because the KH domains do not bind the defined sequences. Taken together, the results of these assays suggest that there are two distinct modes of RNA binding by MTH1203; one through the MBL domain and the other through the KHb domain.

### MTH1203 Is a Zinc-Dependent Nuclease

**Figure 6:**
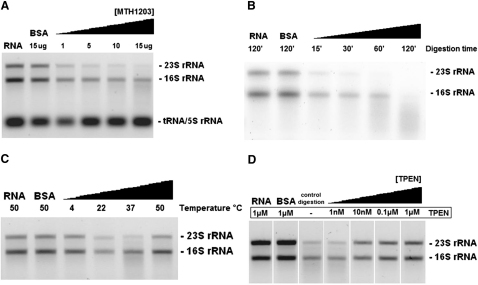
MTH1203 Has Zn-Dependent Nuclease Activity against *E. coli* rRNAs RNA degradation is observed as a decrease in the amount of 16 S and 23 S rRNAs with 1 μg of RNA used per lane. Control lanes contained either RNA with buffer or BSA instead of MTH1203, where the BSA concentration was equal to the highest concentration of MTH1203 in each gel. (A) Degradation assays performed at 37°C with increasing amounts of MTH1203. (B) Degradation assays performed at 37°C with different incubation times (15 μg of MTH1203 per lane). (C) Degradation assays performed at different temperatures (10 μg of MTH1203 per lane). (D) Inhibition of the nuclease activity by incubation with increasing concentrations of TPEN (10 μg of MTH1203 per lane). See also Figure S5.

MTH1203 is predicted to function as an endonuclease and/or an exonuclease as it is homologous to the exonuclease TTHA0252, as well as to the endo- and exonucleases CPSF-73 and RNase J (de la Sierra-Gallay et al., 2008; Ishikawa et al., 2006; Yang et al., 2009). We tested the MTH1203 nuclease activity in vitro against an *Escherichia coli* RNA extract by monitoring the degradation of rRNAs. MTH1203 preferentially degraded the *E. coli* 23S rRNA (2904 bases) and to a lesser extent the 16S (1541 bases) rRNA, but not the 5S rRNA (120 bases) or tRNAs (75 bases) (Figures 6A and 6B). Similarly, TTHA0252 is unable to degrade short RNAs from *E. coli* in vitro (Ishikawa et al., 2006). These experiments do not allow us to conclude whether MTH1203 is an endo- or an exonuclease or whether degradation occurs from the 3′ or the 5′ end. In vitro MTH1203 was shown to be most active at 37°C and at room temperature (22°C), whereas at 4°C and 50°C very little activity was observed (Figure 6C). The poor activity at 50°C was unexpected given that the optimal growth temperature for *M. thermoautotrophicus* is ∼65°C and the protein was stable at 65°C during purification. However, other cellular components could modulate MTH1203 activity in vivo.

Zinc was observed in the active site of the MTH1203 structure and other β-CASP proteins require zinc ions for activity (de la Sierra-Gallay et al., 2008; Ishikawa et al., 2006; Mandel et al., 2006). To test the dependence of the MTH1203 nuclease activity on zinc ions, rRNA degradation experiments were performed in the presence of N,N,N′,N′-Tetrakis-(2-pyridylmethyl)-ethylenediamine (TPEN), a specific chelator of zinc ions. The nuclease activity of MTH1203 was inhibited by TPEN (Figure 6D), and the extent of inhibition was proportional to the TPEN concentration. This result shows that the nuclease activity of MTH1203 depends on the presence of coordinated zinc ions, similar to other members of the MBL superfamily (Bebrone, 2007).

### MTH1203 Is Likely to Be a Dimer In Vivo

To determine the oligomeric state of MTH1203, we performed size exclusion chromatography in line with multiangle laser light scattering. In the presence of 0.5 M NaCl, or 0.7 M NaCl or KCl, MTH1203 eluted as a single peak corresponding to a dimer (Figure S4). However, during purification in the presence of 1 M NaCl MTH1203 eluted from size exclusion chromatography as a single peak with an apparent molecular weight of ∼70 kDa, which corresponds to a monomer (predicted molecular mass of 73.1 kDa, data not shown). Given that intracellular K^+^ levels in *Mth* vary between 0.62 and 0.78 M through different growth phases (Sprott and Jarrell, 1981), it is likely that MTH1203 is a dimer under physiological conditions.

### MTH1203 Is a Specific β-CASP RNase that Functions as a Dimer

The MBL and β-CASP domains of MTH1203 are highly similar to the respective structures of human CPSF-73 (31% sequence identity and an rsmd of 1.8 Å over 421 Cα atoms) and the bacterial protein TTHA0252 (30% sequence identity and an rmsd of 2.4 Å over 427 Cα atoms) (Figure 2C). The most prominent difference between MTH1203 and CPSF-73 or TTHA0252 is the presence of the KH domains at the N terminus of MTH1203, suggesting that MTH1203 and its orthologs might target RNA substrates more specifically than CPSF-73 and TTHA0252.

**Figure 7:**
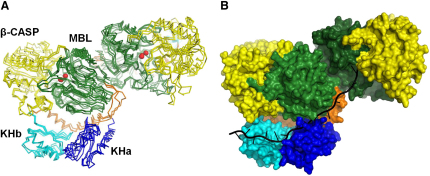
Dimerization of MTH1203 Is Conserved and Suggests a Model for Degradation (A) Dimer of MTH1203 superposed with those of its archaeal orthologs Mm0695 (PDB ID 2xr1) and PH1404 (PDB ID 3af5). (B) Model of a single-stranded RNA binding to the MTH1203 dimer. Domains are colored as in Figure 2. See also Figures S4 and S5.

Analysis of MTH1203 using PISA (Krissinel and Henrick, 2007) indicates that MTH1203 forms a stable dimer in solution. This assembly, with a protein-protein interface area of 933 Å^2^, involves a symmetrical interaction between the C terminus region of each monomer, a loop and a β strand from the MBL domain. The amino acid sequence of the C terminus that forms the interaction interface is highly conserved (Figure 1), suggesting that this dimerization is functionally relevant. Notably, the MTH1203 dimer superposes with the dimers from the recently characterized *P. horikoshii* and *M. mazei* orthologs with rmsds of 1.36 (over 610 Cα atoms) and 1.42 Å (over 590 Cα atoms), respectively (Figure 7A).

We hypothesize that dimerization plays an important role in the function of this RNase. As the oligomeric state of MTH1203 changes depending on the salt concentration, we tested its ability to degrade RNA at different salt concentrations. More RNA was degraded at 0.7 M or lower NaCl, when MTH1203 is dimeric, than compared to that at 1 M NaCl, suggesting that the dimer of MTH1203 is at least 15% more active than its monomeric form (Figure S5). The characterization of MTH1203 as a dimer in solution under physiological conditions and that the dimer enhances activity are both compatible with the model of Mir-Montazeri et al. (2011) in which the KH domain of one monomer binds to an RNA molecule, targeting it for degradation by the MBL-β-CASP domains from the other monomer.

### MTH1203 Activity Might Be Dependent on Structural Plasticity

The two detected activities of MTH1203, that of an RNA-binding protein and that of a nuclease, are expected to be related. Under the model of Mir-Montazeri et al. (2011) the RNA-binding domain from one molecule would initially recognize the RNA substrate that would then be cleaved at the active site of the other subunit suggesting coordinated conformational changes. To examine the elasticity of MTH1203, and potential conformational changes during substrate binding to KH domains and access to the nuclease active site, we performed normal mode analysis calculations using the Webnm@ server. This analysis yielded the prediction that the major movements in MTH1203 are associated with the KH domains (residues 1–138) and the β-CASP domain (residues 384–577), whereas the core MBL domain (residues 180–383) is quite rigid (Figure S6). The linker region (residues 139–179) between the KHb and the MBL domain is also predicted to be flexible. These flexible regions also have relatively high B factors. The observed and predicted mobility are consistent with a coordinated mechanism for RNA degradation. Thus, a ssRNA could be initially recognized by the KH domain(s) from one monomer and, once bound, the β-CASP domain from the second subunit of the dimer would adjust its conformation allowing the RNA molecule to enter the active site targeting it for degradation.

### No Interaction between MTH1203 and the Exosome

To detect protein-protein interactions between MTH1203 and several MTH exosomal proteins, mixtures of proteins were analyzed by SEC-MALLS. MTH680, MTH685, MTH689, MTH1318 (data not shown), and the exosome core, formed by the MTH682 and MTH683 proteins (Ng et al., 2010), were mixed with MTH1203 (Figure S7). No higher molecular weight species, or change in the MTH1203 elution profile, were observed upon mixing with exosome components. Thus, MTH1203 does not appear to directly interact with the exosome.

Pull-down experiments were performed to determine if there was an association between MTH1203 and any soluble protein from *M. thermautotrophicus* cells. The pull-down experiment (Figure S8) revealed a low level of nonspecific interaction between the cell lysate and the resin in the absence of MTH1203 (negative control). No difference compared with the background was observed when MTH1203 was used in the pull-down suggesting that under these conditions MTH1203 did not bind any abundant soluble protein in the cell lysate.

## Discussion

MTH1203 is the *M. thermautotrophicus* representative of a highly conserved set of orthologous archaeal proteins that, by sequence analysis and comparative genomics, has been predicted to possess RNase activity (Koonin et al., 2001). The original comparative-genomic study predicted the components of the archaeal exosome (Koonin et al., 2001). Most of the (predicted) exosome components are encoded within a superoperon that also encodes the catalytic subunits of the archaeal proteasome, suggestive of a functional association between the exosome and proteasome in Archaea (Koonin et al., 2001). MTH1203 was identified as a predicted nuclease whose genomic context, adjacent to a proteasome subunit gene similar to the genomic associations of some exosome components, is conserved in almost all archaeal genomes. On the basis of this conserved genomic context, Koonin et al. (2001) proposed that MTH1203 might be an exosome component or could functionally complement the exosome activity. To test this hypothesis, we solved the structure of MTH1203 and characterized its activity and interactions.

The MTH1203 crystal structure is a novel addition to the β-CASP family that contains additional RNA-binding KH modules. KH domains appear in proteins either as a single copy or as multiple copies with binding affinities for nucleic acids varying from the micromolar to the nanomolar range. For example, the KH domains from Poly(C)-binding protein 1, SF1 and hnRNP K bind their cognate RNAs with dissociation constants of 4.37, 1, and 1.8 μM, respectively (Backe et al., 2005; Liu et al., 2001; Sidiqi et al., 2005). The presence of multiple, repeated KH domains typically increases the affinity toward RNA (Adinolfi et al., 1999; Valverde et al., 2008). Linking several KH domains within the same polypeptide molecule also contributes to affinity, as demonstrated for the K-homology splicing regulator protein that bound RNA more weakly when the KH domains were expressed separately (Garcia-Mayoral et al., 2007).

Structural analysis revealed that MTH1203 contains two KH domains instead of the single KH domain predicted by sequence analysis. The presence of the RNA-binding domains suggests that MTH1203 could more specifically target RNA substrates than other β-CASP proteins that only contain the MBL and β-CASP domains. The KHa domain of MTH1203 and its orthologs does not contain the RNA-binding motif GXXG. The evolutionary conservation of the KHa domain suggests a distinct function, either binding RNA in a noncanonical fashion or an alternative structural or regulatory role.

Fluorescence polarization anisotropy experiments demonstrated that the full-length MTH1203 has the ability to bind ssRNAs with at least a micromolar affinity. The individual KH domains did not bind to the specific RNA sequences tested, but a pull-down assay showed that the KHb domain binds to a degenerate RNA sequence, whereas the KHa-b and KHa RNA binding was reduced, relative to KHb binding, by 56% and 75%, respectively. Of the sequences tested, the full-length MTH1203 interacted primarily with U-rich sequences. Because the specific RNA sequences did not interact with the KH domains, this interaction is likely to be mediated by the MBL domain. However, the MTH1203 MBL domain seems to be specific for U-rich rather than poly(U) sequences because the dissociation constant was lower for U_4_C_4_ and C_4_U_4_ then for U_7_. These experiments show that MTH1203 binds ssRNA but the precise sequences targeted by the KH domains remain to be determined.

The model of the MTH1203-KHb domain complex with RNA does not provide insight into the orientation of the RNA substrate in the catalytic site of the same subunit of the dimer because of the substantial ∼42 Å spatial separation, at least ∼10 nucleotides apart if both sites are bound to an extended RNA molecule. However, the indications that MTH1203 functions as a dimer raise the possibility that an RNA molecule binds to the KH domain from one subunit of the dimer and is cleaved by the MBL-β-CASP of the other subunit (Mir-Montazeri et al., 2011). While the spatial separation is larger (∼73 Å), this configuration is geometrically favorable with the bound RNA directed through the groove formed between the KHa and MBL domains from one subunit into the active site on the second subunit (Figure 7B). A positive charge is visible on the molecular surface along this groove (Figure S1) suggesting that it could accommodate RNA. Our data showing that MTH1203 is more active at salt concentrations at which it forms a dimer support this mechanism.

The MTH1203 MBL and β-CASP domains, including the active site, are highly similar to those already characterized in CPSF-73 and TTHA0252, suggesting that the mechanisms of RNA hydrolysis could be the same. Although there is no structure of a β-CASP protein in complex with nucleic acids, a mechanism of RNA cleavage has been proposed for RNase Z, an MBL-family but non-β-CASP endoribonuclease (de la Sierra-Gallay et al., 2005), based on that for human glyoxalase II (Cameron et al., 1999). In this mechanism, Asp67 of RNase Z (coordinated to Zn1) removes a proton from a water molecule, generating a hydroxide, which makes a nucleophilic attack on the phosphate molecule. Mth1203 contains Asp246 in an equivalent position suggesting that it plays a similar catalytic role.

Preliminary nuclease activity assays on the degradation of *E. coli* rRNAs confirmed that MTH1203 has a metal-dependent nuclease activity. In vitro MTH1203 completely degraded the 23S and 16S rRNAs. However, no activity was observed against the short *E. coli* 5S rRNA or tRNAs. As rRNAs possess extensive secondary structure, this result suggests that MTH1203 degrades substrates independent of their structure. It remains to be determined whether MTH1203 has an endo- and/or exonuclease activity. Additionally, characterization of the sequence specificity of MTH1203, both of the nuclease and RNA binding functions, will provide insight into its in vivo function.

In summary, we present experimental evidence in support of the hypothesis that MTH1203 could function alongside the exosome in RNA degradation (Koonin et al., 2001). The possibility that MTH1203 is an exosome subunit was not supported by our protein-protein interactions experiments. However, the nuclease activity of MTH1203 decreased when temperature was increased from 37°C to 50°C. This could mean that in vivo, at the optimum growth temperature of 65°C, MTH1203 would need other factors to function. No protein interactions were revealed between MTH1203 and any other MTH protein in our pull-down assays or using SEC-MALLS to study targeted mixtures. However, a functional relationship does not necessitate formation of stable complexes and could be transient or mediated by other factors. We propose that the novel combination of KH domains with the MBL domain found in MTH1203 specifically targets cellular RNAs for degradation, possibly functioning in concert with the exosome.

## Experimental Procedures

### Cloning, Protein Expression, and Purification

The *mth1203* gene from the delta H strain of *Methanothermobacter thermautotrophicus* was amplified by PCR from genomic DNA and cloned using a ligase-independent reaction into the vector pETYSBLIC-3C (Fogg and Wilkinson, 2008). The KH domains of MTH1203 (KHa [3–68], KHb [72–136], and KHa-b [3–136]) were cloned between the NdeI and XhoI restriction sites in pET28 (Novagen). These constructs were expressed in *E. coli* BL21 (DE3) Rosetta expression cells grown in Terrific Broth at 37°C. The seleno-methionine (SeMet) derivative of MTH1203 was expressed in the same cells grown at 37°C in autoinduction media (Sreenath et al., 2005) with overnight incubation at 30°C.

The MTH1203 proteins were purified at room temperature. Cell pellets were resuspended in 50 mM KH_2_PO_4_ (pH 7.5), 1 M NaCl, 2 mM MgCl_2_, and 10 mM imidazole and lysed by sonication and then the His-tagged protein was purified from the clarified lysate using a 5 ml HiTrap nickel-chelating column (GE Healthcare). The protein was then purified using a Superdex 200 gel filtration column (GE Healthcare) after removal of the His-tag with 3C protease. The purified protein was concentrated to 10 mg ml^-1^ for crystallization trials.

For complete details of the cloning, expression, and purification see the Supplemental Experimental Procedures.

### Crystallization

Preliminary crystallization screening was performed using a Mosquito nanoliter pipetting robot (TTP Labtech) to set up 96-well sitting drop plates at room temperature, with 150 nl of MTH1203 mixed with 150 nl of reservoir solution. Crystals were initially optimized robotically then scaled up using hanging-drop vapor diffusion in 24-well plates with a 1:1 mixture of protein solution and reservoir solution (1 μl each). For diffraction data collection, a single brick-shaped crystal was grown over 24 hr in 50 mM KH_2_PO_4_ [pH 5.2], 2% PEG 2000 MME, 7% ethylene glycol, 0.5 mM ZnCl_2_, 1 mM tris(2-carboxyethyl)phosphine (TCEP), 10 mM D-Ribose-5-phosphate (Fluka). This crystal was flash-cooled in liquid nitrogen without further cryoprotection. SeMet protein crystals were grown in 24-well plates in identical condition to the native crystals, containing 5 mM DTT.

### Data Collection, Structure Determination, and Refinement

Single-wavelength anomalous dispersion (SAD) data were collected to 3.1 Å at the zinc peak (1.2820 Å), at the ID29 station at the ESRF. These data were processed with *DENZO* and *SCALEPACK* (Otwinowski and Minor, 1997) and solved by molecular replacement in P4_1_2_1_2 using the program *BALBES* (Long et al., 2008) with the coordinates of the human CPSF-73 (PDB code 2i7v) as the search model. Two molecules were located in the asymmetric unit, corresponding to a solvent content of 72%. *SHARP* (Abrahams and Leslie, 1996; Fortelle and Bricogne, 1997; Vonrhein et al., 2007) and *DM* (Cowtan and Zhang, 1999) were used for density modification. The initial structure was built using *BUCCANEER* (Cowtan, 2006) and improved with cycles of model building using *QUANTA* (Accelrys Inc., San Diego, CA) and refinement using *REFMAC* (Murshudov et al., 1997) with TLS and NCS. *PROCHECK* (Laskowski et al., 1993) was used for structure validation. SAD data of the SeMet crystals were collected to 3.5 Å at the selenium peak (0.9739 Å), at ID29 station at the ESRF and were processed in the same way as the data collected at the Zn peak.

### RNA Degradation Assays

Total RNAs purified from *E. coli* Nova Blue cells were used as the substrate for nuclease activity experiments. The reaction buffer contained 10 mM Tris (pH 8.0), 0.2 mM DTT, 2 mM MgCl_2_, 0.1 mM EDTA, and 0.5 M NaCl except to test the inhibition of the protein nuclease activity when MTH1203 was incubated in 10 mM Tris (pH 8.0), 0.2 mM DTT, 0.1 mM EDTA, 0.5 M NaCl, and N,N,N′,N′-Tetrakis-(2-pyridylmethyl)-ethylenediamine (TPEN). Before each reaction, the protein was incubated in the reaction buffer for 10 min at 37°C prior to RNA addition and subsequently incubated for 90 min at 37°C or at the indicated temperatures/times. For the nuclease inhibition assays MTH1203 was incubated prior to RNA addition for 30 min at 37°C. Bovine serum albumin (BSA) was always used in the same conditions as MTH1203 as a control. Unless otherwise indicated, RNA from the samples was then isolated by phenol/chloroform extraction and loaded on a 1% agarose gel stained using SYBRsafe (Invitrogen). The relative activities of the monomer and dimer were estimated by analyzing the gel using ImageJ (U.S. National Institutes of Health, Bethesda, MD).

### Fluorescence Polarization Anisotropy Measurements

Eight different single-stranded RNA fragments labeled with a 5′ fluoresceine were synthesized by Dharmacon (A_7_, G_7_, C_7_, U_7_, C_4_U_4_, U_4_C_4_, A_4_G_4_, and a degenerate A_2_X_4_G_2_). The fluorescence anisotropy experiments were performed in a Spex Fluoromax-3 (Horiba Jobin Yvon), with excitation and emission wavelengths of 485 and 520 nm, respectively. Experiments were performed in 20 mM Tris (pH 8.0) and 1 M NaCl. In each titration, His-tagged constructs of MTH1203 at 1 mg ml^-1^ were added to RNA (5 nM). After mixing for 1 min, three measurements were read with a 5 s integration. Control experiments were also performed in the same conditions where BSA was used instead of MTH1203. Titration curves were fit using Scientist (Micromath).

### Pull-Down Assays

*M. thermautotrophicus* cell pellets were resuspended in 2 ml of buffer (500 mM KOAc, 20 mM Tris [pH 8.0], 20 mM imidazole, 1 mM EDTA, and 1 mM DTT), lysed by sonication and then cleared by centrifugation at 15,000 × g for 15 min. MTH1203 (0.15 mg with hexahistidine tag) was bound to 15 μl of chelating Sepharose fast flow resin (GE Healthcare) charged with Ni^2+^ equilibrated in the lysis buffer. The resin was then washed and resuspended in buffer to give a total volume of 100 μl. Ten microliters of the resin was added to 400 μl of either the cell lysate supernatant (experimental sample) or buffer (binding control). A control using resin without bound MTH1203 was also performed. After 30 min each sample was washed with 2 × 400 μl of buffer, resuspended in 10 μl of 4 × SDS-PAGE loading buffer, boiled, and run on a 12% SDS-PAGE gel stained with Coomassie blue.

## Figures and Tables

**Table 1:** X-Ray Data Collection and Refinement Statistics

Data Collection	Native (Zn Peak)	SeMet (Se Peak)
Wavelength (Å)	1.2820	0.9739
Resolution range (Å)	30–3.10 (3.21–3.10)	50–3.50 (3.63–3.50)
Space group	P4_1_2_1_2	
Unit cell parameters (Å, °)	*a* = 112.0, *c* = 400.6	*a* = 112.5, *c* = 402.0
Number of unique reflections	46,893 (4283)	33,316 (3130)
Completeness (%)	99.3 (93.1)	98.8 (94.7)
Redundancy	11.0 (7.7)	5.5 (5.1)
R_merge_^a^ (%)	11.9 (60.4)	9.9 (27.7)
I/σ (I)	17.9 (2.0)	13.1 (3.3)
Wilson B (Å^2^)	96.5	55.8

**Refinement**		

Resolution (Å)	3.1	
R-factor^b^ / R_free_^c^	0.2146/0.2508	
Number of free reflections	957	
No. nonhydrogen atoms	10,285	
No. water molecules	28	
No. zinc atoms	5	
No. phosphate ions	4	
Rmsd (bond distance) (Å)	0.0076	
Rmsd (bond angle) (deg)	0.9562	
Average B value (Å^2^)	105.2	
Ramachandran plot		
Most favored region (%)	87.6	
Additional allowed regions (%)	12.1	
Generously allowed regions (%)	0.2	
Disallowed regions (%)	0.2	

Values in parentheses represent data for the highest resolution shell.

a R_merge_ = Σ *| I_i_ –* < *I* > | / Σ < I > , where *I* is the measured intensity of each reflection and *< I >* is the intensity averaged from multiple observations of symmetry-related reflections.

b R-factor = Σ *|F*_obs_*| –* |*F*_calc_| / Σ |*F*_obs_|, where *F*_obs_ and *F*_calc_ are the observed and the calculated structure-factor amplitudes, respectively.

c R _free_ is the *R*-factor calculated with 2% of the reflections chosen at random and omitted from refinement.

## Accession Numbers

Atomic coordinates and structure factors were deposited in the Protein Data Bank under the ID code 2ycb.

## Supplemental Information

Document S1. Eight Figures and Supplemental Experimental Procedures
