# Supplementary material for: Structure and Activity of a Novel Archaeal β-CASP Protein with N-Terminal KH Domains
Source: Structure. 2011 May 11;19(5):622–32. doi: 10.1016/j.str.2011.03.002 (PMC3095777; doi:10.1016/j.str.2011.03.002)
Supplement: Document S1. Eight Figures and Supplemental Experimental Procedures [file mmc1.pdf]

Supplemental Information

Structure and Activity of a Novel Archaeal β-CASP Protein with N-Terminal KH Domains

Ana P.G. Silva, Maria Chechik, Robert T. Byrne, David G. Waterman, C. Leong Ng, Eleanor J. Dodson, Eugene V. Koonin, Alfred A. Antson, and Callum Smits

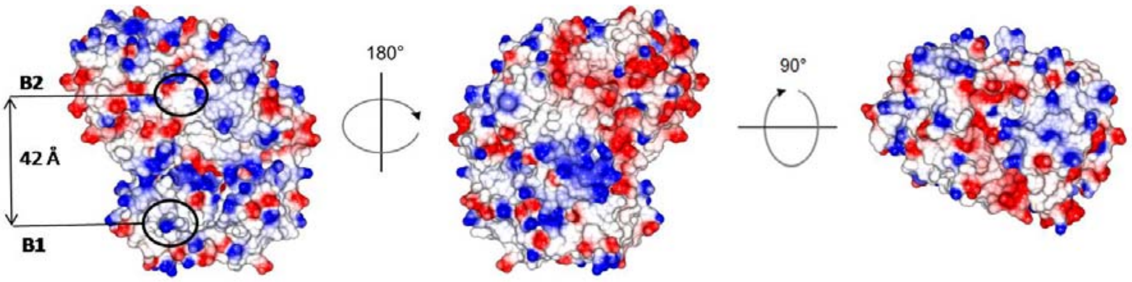

Figure S1, related to Figure 2: The surface of MTH1203 coloured by electrostatic potential. The initial orientation is the same as in the Figure 2B. The surface locations of the KH2 RNA binding site (B1) and the nuclease active site (B2) are indicated.

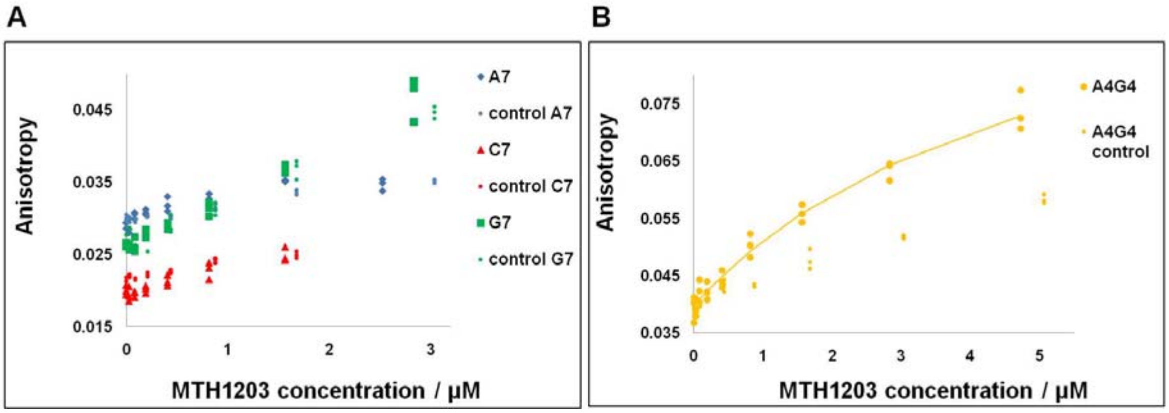

Figure S2, related to Figure 5: MTH1203 polarisation anisotropy experiments with different RNA sequences. The change in fluorescence polarisation anisotropy of fluoresceine-labeled RNAs was measured using increasing MTH1203 concentrations with BSA as a negative control. (A) Titration of A_7, C_7 and G_7 showed no evidence of binding to the ssRNA since it presents the same result as the BSA control. (B) Titration of A_4G_4 indicated an interaction with a calculated K_d of 5.3 µM. Calculation of the dissociation constants using Scientist (Micromath) indicated a good fit of the data, with the residual errors associated with the calculations of 4% or less (data not shown). Data points correspond to multiple measurements from the same titration.

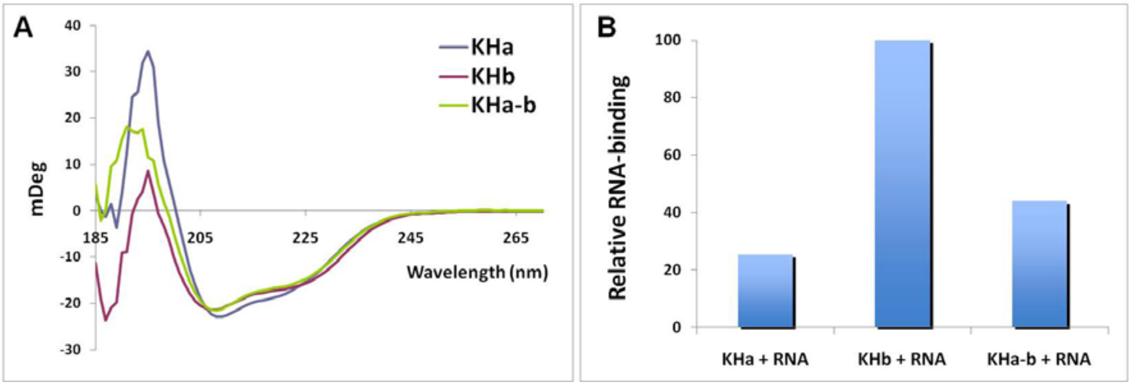

Figure S3, related to Figure 5: KHb is a more active RNA-binding domain than KHa. A) Circular Dichroism spectra of the KH domains in 20 mM Na Phosphate pH 7.5 and 50 mM NaCl. B) Quantitation of the fluorescence after a pull-down using the different KH domains to bind the degenerate RNA sequence, $A_2X_4G_2$. The binding results were detected by reading the fluorescence of the pull-down fractions and have background binding subtracted, and are normalised to the fluorescence obtained for the KHb domain.

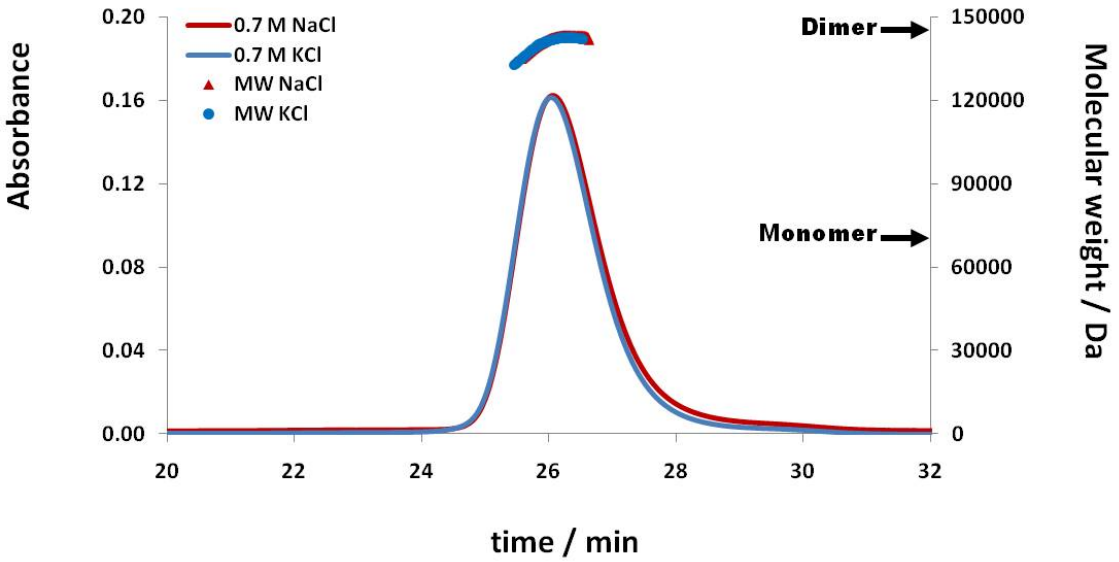

Figure S4, related to Figure 7: MTH1203 is a dimer. SEC-MALLS results show that the molecular weight of MTH1203 in solution, containing 0.7 M NaCl or KCl, is around 145 KDa.

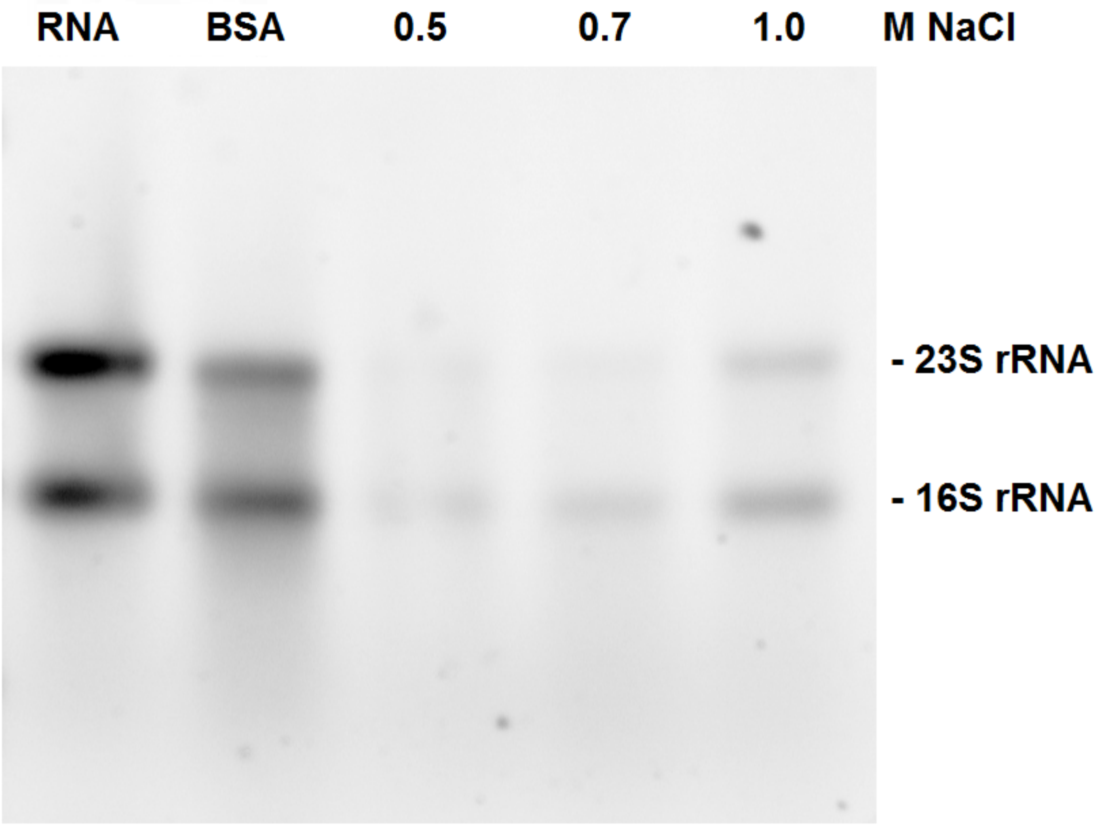

Figure S5, related to Figures 6 and 7: MTH1203 is a more active nuclease as a dimer. RNA digests were performed using MTH1203 in different salt concentrations. MTH1203 is a dimer in both 0.5 M and 0.7 M NaCl, but a monomer in 1 M NaCl.

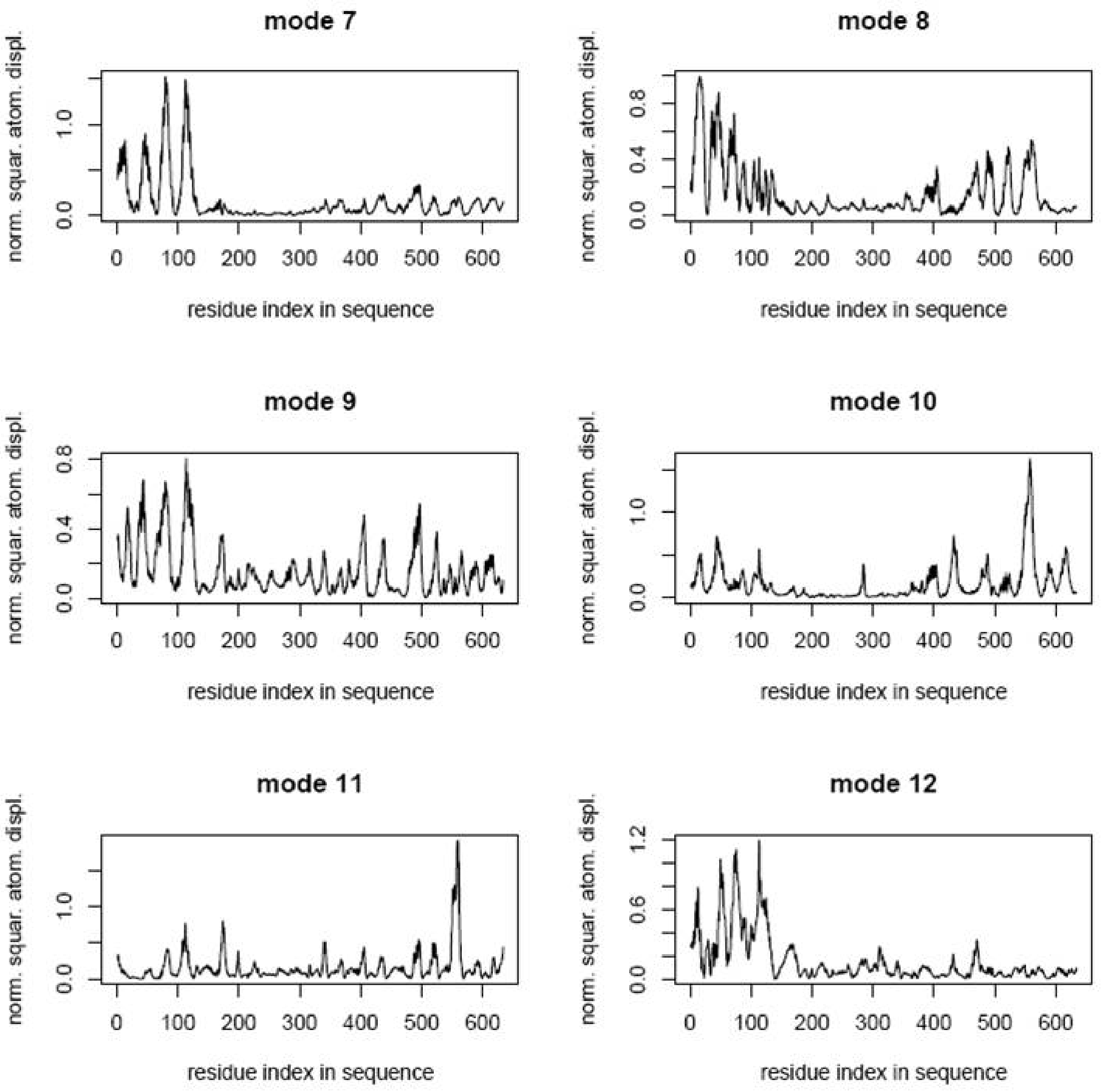

Figure S6, related to Results: Flexibility of MTH1203. Plot of the normalized squared atomic displacement for each residue of chain A of MTH1203 for the low frequency normal modes. The plot of displacements for each Cα atom highlights which parts of the protein are the most mobile for each mode. Analysis performed by the WEBnm@ from the Norwegian Bioinformatics platform (http://services.cbu.uib.no/tools/normalmodes).

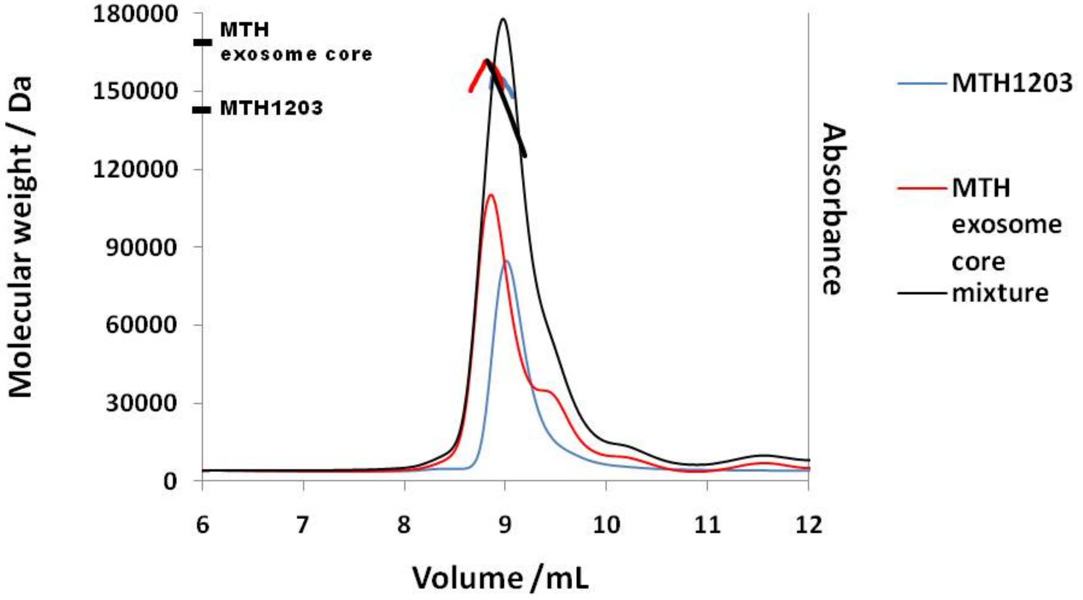

Figure S7, related to Results: SEC-MALLS experiments of MTH1203 and the MTH exosome core (MTH682/MTH683). The expected molecular weights for the MTH1203 dimer and MTH1203 exosome core are indicated. In these experimental conditions (20 mM Tris pH 7.5 and 0.5 M NaCl) there is no detectable interaction between MTH1203 and the exosome core.

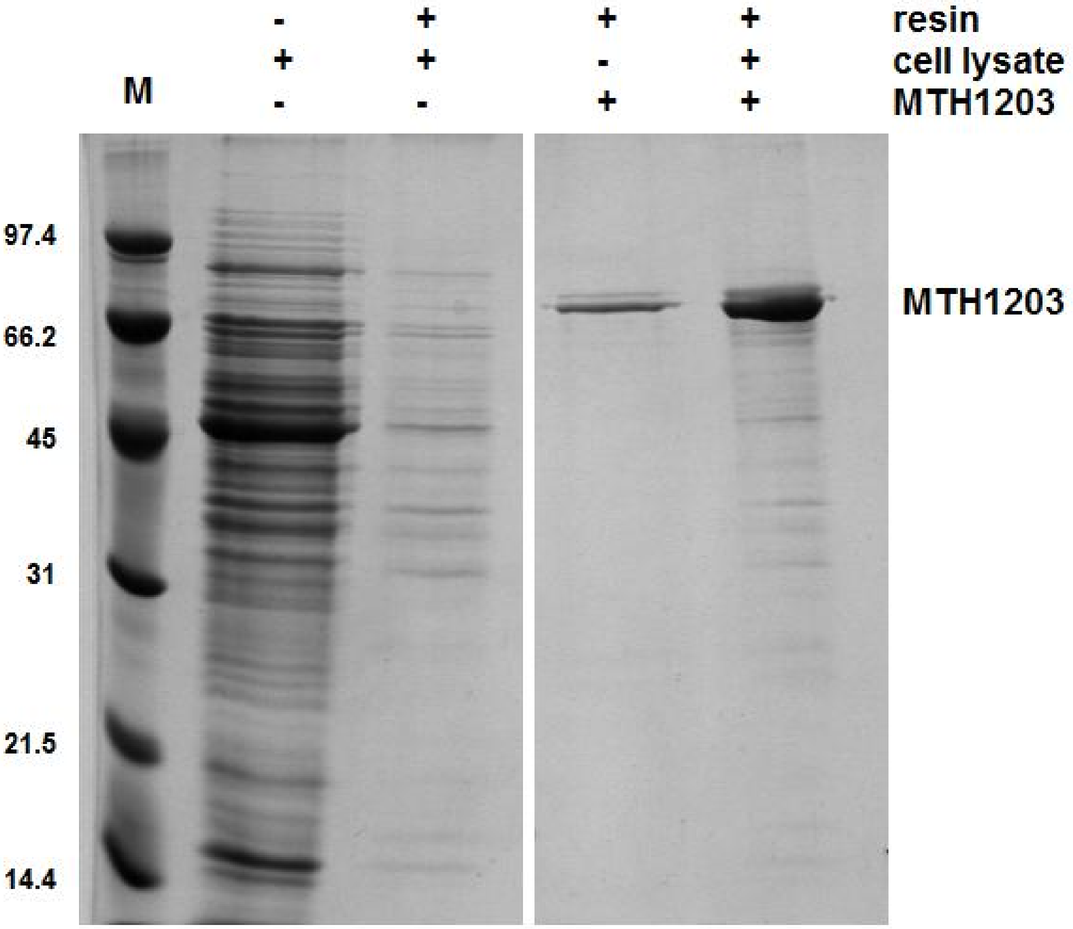

Figure S8, related to Results: Interaction assay using MTH1203 to pull-down proteins from a *M. thermautotrophicus* cell lysate. MTH1203 with a hexahistidine-tag was bound to a Ni^{2+} resin and incubated with MTH cell lysate. No proteins from the MTH lysate were observed to interact with Nickel-resin bound MTH1203.

Supplemental Experimental Procedures

Cloning and protein expression

The β-CASP protein predicted to be functionally related to the exosome is encoded by the gene *mth1203* in the strain delta H of *Methanothermobacter thermautotrophicus*, was amplified by PCR from genomic DNA. The oligonucleotides **CCAGGGACCAGCA**ATGGTTTCAGAGATGCTTGAAGAAATCAAAAGGAC and **GAGGAGAAGGCGCGTTA**TCACTGGATACGCACGGTTTCCAGG were synthesised (Eurofins MWG Operon) and used as forward and reverse primers, respectively, incorporating sequences (in bold) with compatible overhangs to the vector pETYSBLIC-3C (Fogg and Wilkinson, 2008). The amplified fragment was then annealed to petYSBLIC-3C in a ligase-independent reaction (Fogg and Wilkinson, 2008) and confirmed by sequencing. The KH domains of MTH1203 (KHa (3-68), KHb (72-136) and KHa-b (3-136)) were amplified from the plasmid DNA of the full length MTH1203. All constructs were digested with restriction enzymes NdeI and XhoI and ligated into pET28, which was similarly digested.
These constructs, which encode N-terminal hexa-histidine tagged proteins, were used to transform *E. coli* BL21 (DE3) Rosetta expression cells and grown at 37 °C in Terrific Broth media (1.2% w/v tryptone, 2.4% w/v yeast extract, 0.4% v/v glycerol and 1.21% w/v KH_2PO_4, pH 7.5) containing kanamycin (30 µg ml^{-1}) until an OD_{600} of 0.6-0.8. Protein expression was induced by adding isopropyl thio-β-D-galactopyranoside to a final concentration of 1 mM and the culture was harvested after overnight incubation at 30 °C. The seleno-methionine (SeMet) derivative of MTH1203 was expressed in *E. coli* BL21 (DE3) Rosetta pLysS cells grown at 37 °C in auto-induction media (Sreenath et al., 2005) containing kanamycin (30 µg ml^{-1}) and chloramphenicol (30 µg ml^{-1}) until an OD_{600} of 2.0, after which the cells were incubated overnight at 30 °C.

Protein purification

Protein purification was performed at room temperature, since the protein precipitates at low temperatures. Cell pellets were resuspended in 50 mM KH_2PO_4 pH 7.5, 1 M NaCl, 2 mM $MgCl_2$ and 10 mM imidazole (5 ml buffer per gram of cells) and then disrupted by sonication. The lysate was incubated at 65 °C for 30 min, centrifuged at 27,000 x g for 45 min and the supernatant cleared through a 0.45 µm filter (Millipore). The clarified protein extract was loaded onto a 5 ml HiTrap nickel-chelating column (GE Healthcare) and eluted by a linear gradient to 500 mM imidazole. Fractions containing the protein were pooled, buffer exchanged to remove imidazole, concentrated until 2 mg ml^{-1} and digested with 3C protease (1:50) to cleave the hexa-histidine tag, which was subsequently removed using a 5 ml HiTrap column. Protein fractions were concentrated and loaded onto a Superdex 200 gel filtration column (GE Healthcare) equilibrated in 50 mM KH_2PO_4 pH 7.5, 1 M NaCl and 2 mM $MgCl_2$. The purified protein was concentrated to 10 mg ml^{-1} for crystallisation trials. Purification of the KH domains and the SeMet derivative was performed in similar way to the native protein, although the hexa-histidine tag was not cleaved.

Fluorescent pull-down assay

For the fluorescence pulldown assays, 1 µM of the fluorescein-tagged degenerate $A_2X_4G_2$ oligo was added to 100 µl of his-tagged protein at 0.5 mg/ml in 20 mM Tris pH 8.0 with 150

mM NaCl. This mixture was incubated in the dark for 20 min at room temperature. 20 µl of Ni^{2+} resin slurry equilibrated in binding buffer was added and mixed for a further 20 min in the dark. The resin was pelleted and washed three times and then bound protein or protein/RNA complex was eluted in 500 µl binding buffer with 0.4 M imidazole. The fluorescence in each sample was quantitated using a Spex Fluoromax-4 (Horiba Jobin Yvon) at 25 ˚C with excitation and emission wavelengths of 485 and 520 nm respectively with a 2 s integration time. For each protein a control was performed without RNA added. This background fluorescence was subtracted from the RNA bound sample to provide the final reading.

SEC-MALLS experiments

MTH1203 and other predicted exosome subunit proteins (MTH680, MTH685, MTH689, MTH1318 and the exosome core MTH682/MTH683) were used at 1 mg ml^{-1}. Standalone proteins and 1:1 ratio mixtures of each exosome component with MTH1203 were separated on a BioSep-SEC-S 3000 column (Phenomenex) equilibrated in 20 mM Tris pH 7.5 and 0.5 M NaCl at 1 ml min^{-1}. Light scattering data were recorded on an in-line Dawn Heleos II (Wyatt Technology) with an in-line Optilab rEX refractometer (Wyatt Technology). A refractive index increment (d*n*/d*c*) estimate of 0.183 ml g^{-1} was used for protein in 0.5 M NaCl (Folta-Stogniew and Williams, 1999).

Similar experiments were performed to analyse the oligomerization state of MTH1203. Stand alone protein was separated on a S200 (10/30) column at 0.5 ml min^{-1}, equilibrated in buffer 20 mM Sodium Phosphate pH 7.5, 2 mM $MgCl_2$ and 0.7 M NaCl or KCl.
